# Draft genome sequence data of *Paenibacillus cisolokensis* strain LC2-13A and *Xylanibacillus composti* strain K-13

**DOI:** 10.1016/j.dib.2021.107361

**Published:** 2021-09-08

**Authors:** Ayaka Uke, Chinda Chhe, Sirilak Baramee, Chakrit Tachaapaikoon, Patthra Pason, Rattiya Waeonukul, Khanok Ratanakhanokchai, Akihiko Kosugi

**Affiliations:** a Japan International Research Center for Agricultural Sciences (JIRCAS), Biological Resources and Post-Harvest Division, 1-1 Ohwashi, Tsukuba, Ibaraki 305-8686, Japan; b Graduate School of Life and Environmental Sciences, University of Tsukuba, 1-1-1 Tennodai, Tsukuba, Ibaraki 305-8572, Japan; c Pilot Plant Development and Training Institute (PDTI), King Mongkut's University of Technology Thonburi (KMUTT), Bangkok 10150, Thailand; d Enzyme Technology Laboratory, School of Bioresources and Technology, King Mongkut's University of Technology Thonburi (KMUTT), Bangkok 10150, Thailand

**Keywords:** *Paenibacillus cisolokensis*, *Xylanibacillus composti*, Whole-genome sequencing, Xylanase, Cellulase, Glycosyl hydrolase, GH family

## Abstract

To discover more efficient degradation processes of lignocellulosic biomass, it is still important to analyze genomic and enzymatic data from bacteria that have strong xylanolytic ability. Here, we present the draft genome sequences of the xylanolytic bacteria *Paenibacillus cisolokensis* strain LC2-13A and *Xylanibacillus composti* strain K-13 that are closest to *Paenibacillus* sp. strain DA-C8, which has strong xylan degradation ability under anaerobic growth conditions. Whole-genome sequencing on the Ion GeneStudio S5 System yielded 277 contigs with total size 5,305,208 bp and G+C content 52.3 mol% for strain LC2-13A and 115 contigs with total size 4,652,266 bp and G+C content of 56.2 mol% for strain K-13. The LC2-13A genome had 5,744 protein-coding sequences (CDSs), 57 tRNAs, and 4 clustered regularly interspaced short palindromic repeats (CRISPRs), and the K-13 genome had 4,388 CDSs, 1 rRNA gene, 45 tRNAs, and 5 CRISPRs. The CDSs of LC2-13A and K-13 encoded the following carbohydrate-active enzymes: 98 and 67 glycoside hydrolases, 31 and 29 glycosyl transferases, 23 and 17 carbohydrate esterases, and 13 and 37 carbohydrate-binding modules, respectively. The whole-genome sequences of LC2-13A and K-13 have been deposited in DDBJ/ENA/GenBank under accession numbers BOVK00000000 and BOVJ00000000. The versions described in this paper are version 1.

## Specifications Table

**Table:** 

Subject	Microbiology
Specific subject area	Bacteriology, Genomics
Type of data	Table Figure
How data were acquired	Whole-genome sequencing using the Ion GeneStudio S5 System
Data format	Raw Analyzed
Parameters for data collection	Genomic DNA from pure cultures of strains LC2-13A (DSM 101873) and K-13 (DSM 29793) was extracted and used.
Description of data collection	Sequencing was performed in accordance with Ion GeneStudio S5 System protocols. The CLC Genomic Workbench v.21.0.3 was used for *de novo* assembly, and annotation was carried out using the DDBJ Fast Annotation and Submission Tool (DFAST). Functional annotation was determined using the dbCAN2 server.
Data source location	Institute: Japan International Research Center for Agricultural Sciences (JIRCAS) City/Town/Region: Tsukuba, Ibaraki Country: Japan
Data accessibility	The draft genome sequences of *Xylanibacillus composti* and *Paenibacillus cisolokensis* have been deposited in DDBJ/ENA/GenBank under accession numbers BOVK00000000.1 and BOVJ00000000.1. The direct URLs to the data sets are https://www.ncbi.nlm.nih.gov/nuccore/BOVK00000000 and https://www.ncbi.nlm.nih.gov/nuccore/BOVJ00000000.1/. BioSample and BioProject IDs of *X. composti* in databanks are SAMD00296212 (https://www.ncbi.nlm.nih.gov/biosample/18876272) and PRJDB11487 (https://www.ncbi.nlm.nih.gov/bioproject/725438), and for *P. cisolokensis* the IDs are SAMD00296211 (https://www.ncbi.nlm.nih.gov/biosample/SAMD00296211), and PRJDB11488 (https://www.ncbi.nlm.nih.gov/bioproject/725439).

## Value of the Data

• Genome data from xylanolytic bacteria can be used to design methods for the efficient biological saccharification of lignocellulosic biomass.
• The *Paenibacillus cisolokensis* and *Xylanibacillus composti* genome data can be used to understand the taxonomy and systematics of xylanolytic *Paenibacillus* species.
• The genome data from *P. cisolokensis, X. composti* can be compared with those of closely related *Paenibacillus* species to better understand lignocellulose degradation and improve its efficiency in xylanolytic *Paenibacillus* species.

## 1 Data Description

Plant biomass is composed mainly of three different polymers, i.e., cellulose, hemicelluloses, and lignin. Xylan is the major component of hemicellulose, which is one of the most abundant plant polysaccharides in nature. Endo-β-1,4-xylanases (EC 3.2.1.8) are important glycoside hydrolases that degrade xylan [1]. Many of the characterized xylanases belong to glycoside hydrolase families 10 and 11, according to the classification in the Carbohydrate-Active Enzymes (CAZy) database (http://www.cazy.org). Among the xylanolytic bacteria, species in genus *Paenibacillus* are known to produce a variety of xylan degradation enzymes, such as β-1,4-xylanases, β-xylosidases, and α-L-arabinofuranosidase, with potential applications in industrial manufacturing processes [2]. Here, we present the first draft genome assemblies with annotation data of *Paenibacillus cisolokensis* strain LC2-13A (DSM 101873, NCBI Reference Sequence: NR_151901.1) [3] and *Xylanibacillus composti* strain K-13 (DSM 29793, NCBI Reference Sequence: NR_159901.1) [4], which were isolated from the Cisolok geyser (west Java, Indonesia) and a manure compost pile in Hungary, respectively. *P. cisolokensis* LC2-13A and *X. composti* K-13 are closely related to the thermophilic, facultatively anaerobic, xylanolytic bacterium *Paenibacillus* sp. strain DA-C8 (Accession number: BMAQ00000000.1), as was previously reported [5]. Strain DA-C8 shows strong xylan degradation ability under anaerobic growth conditions. Thus, comparisons of genomic information among these three bacteria will help in understanding the differences in their xylan degradation abilities and properties.

**Fig. 1:**
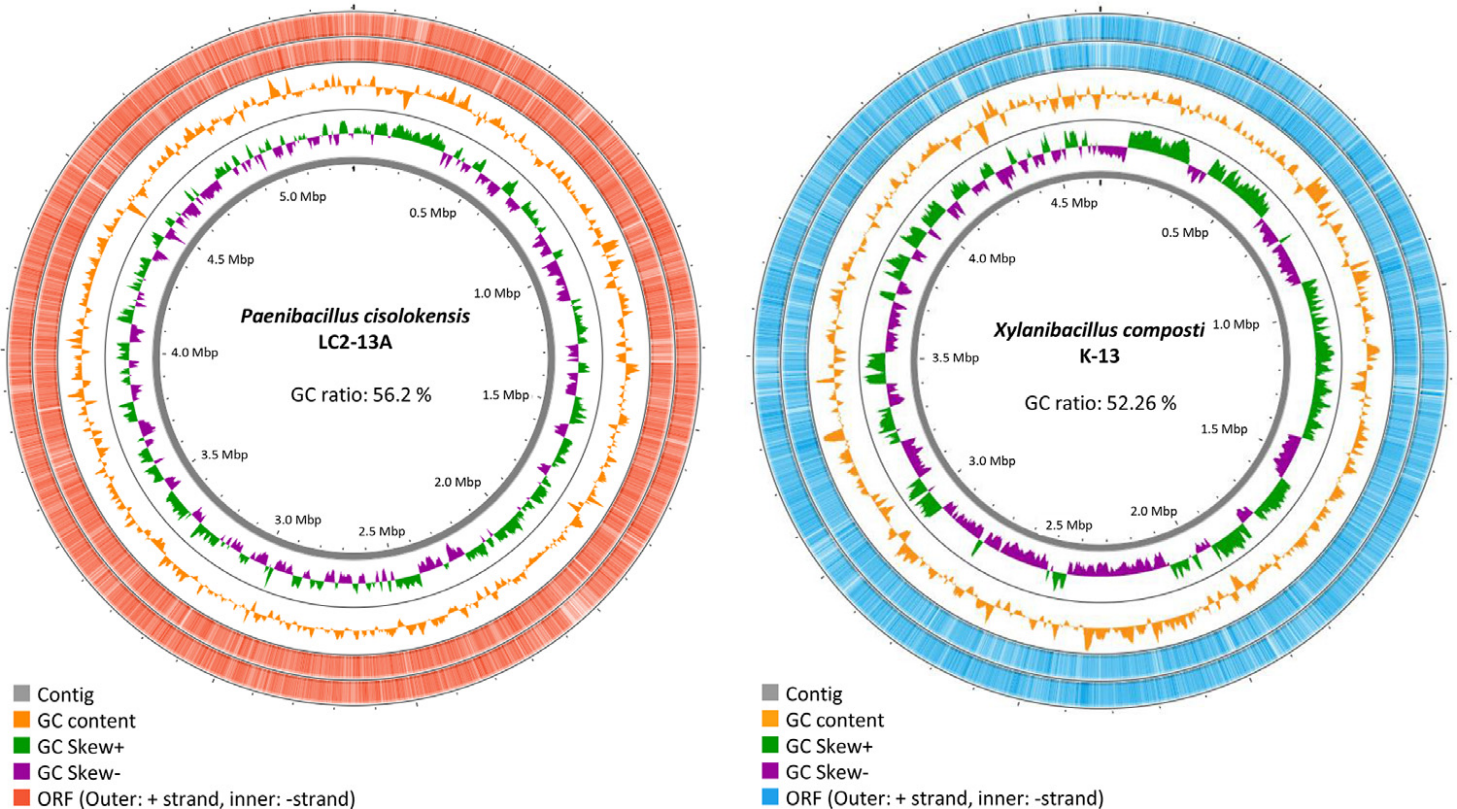
Circular genome map of *Paenibacillus cisolokensis* LC2-13A and *Xylanibacillus composti* K-13. The red and blue outer rings indicate CDS in each DNA direction. The bar graph rings are GC ratio and GC skew. The gray innermost rings are segmented by contig length.

*P. cisolokensis* LC2-13A and *X. composti* K-13 were sequenced and a total of 5,305,208 bp and 4,652,266 bp were obtained, comprising 277 and 115 contigs with G+C content 56.2% and 52.26%, respectively (Fig. 1, Table 1). There were 5,744 protein-coding sequences (CDSs), 57 transfer RNAs (tRNA), and 4 clustered regularly interspaced short palindromic repeats (CRISPRs) for LC2-13A, and 4,388 CDSs, 1 ribosomal RNA, 45 tRNA, and 5 CRISPRs for K-13. The functional gene analysis of the LC2-13A and K-13 genomes detected a total of 165 and 150 genes associated with carbohydrate metabolism, including glycosyltransferases, glycoside hydrolases, carbohydrate-binding modules, and carbohydrate esterases (Table 2).

**Table 1:** Features of the *Paenibacillus cisolokensis* LC2-13A and *Xylanibacillus composti* K-13 genome sequences.

	Strains	
Feature	*P. cisolokensis*	*X. composti*
Number of reads used in the assembly	323,625,816	874,250,486
Genome size	5,305,208	4,652,266
Number of contigs	277	115
G+C content (%)	52.26%	56.20%
Mean contig length	19,152	40,454
Number of CDSs	5,744	4,388
Number of rRNAs	0	1
Number of tRNAs	57	45
Number of CRISPRs	4	5
Genome coverage depth	73x	250x

**Table 2:** Numbers and classes of carbohydrate-active enzymes encoded in the *Paenibacillus cisolokensis* LC2-13A and *Xylanibacillus composti* K-13 genomes.

Enzyme classes	*P. cisolokensis*	*X. composti*
Glycoside hydrolases (GHs)	98	67
Glycosyl transferases (GTs)	31	29
Carbohydrate esterases (CEs)	23	17
Carbohydrate-binding modules (CBMs)	13	37
Total	165	150

## 2 Experimental Design, Materials and Methods

### 2.1 Bacterial strain

LC2-13A was cultured in official DSMZ medium 220:caso agar, which consisted of 15.0 g/L peptone from casein, 5.0 g/L peptone from soymeal, and 5.0 g/L sodium chloride, with pH adjusted to 7.3, and K-13 was cultured in DSMZ medium 92:trypticase soy yeast extract, which consisted of 30.0 g/L trypticase soy broth and 3.0 g/L yeast extract.

### 2.2 Genomic DNA purification and sequencing

The cells were cultivated for 2 days under anaerobic conditions at 45 °C, then genomic DNA was extracted using NucleoBond® AXG Columns and NucleoBond® Buffer Set III (Macherey-Nagel, TaKaRa Bio Inc., Kusatsu, Japan) following the manufacturer's protocols. The quantity and purity of the genomic DNA were determined using a NanoDrop One UV-Vis Spectrophotometer and a Qubit 4 Fluorometer (Thermo Fisher Scientific, Waltham, MA, USA), respectively. To construct the libraries, the genomic DNA was fragmented using an Ion Xpress™ Plus Fragment Library Kit (cat. no. #4471269, Thermo Fisher Scientific) following the manufacturer's protocols. The fragmented libraries, which were approximately 200–300 bp in size, were collected by electrophoresis on Invitrogen E-Gel™ SizeSelect™ II Agarose Gels, 2% (cat. no. #G661012, Thermo Fisher Scientific). Each library was diluted to 25 pM and processed using Ion Chef Systems with the Ion 510, Ion 520, and Ion 530 Kit (cat. no. #A34019, Thermo Fisher Scientific). The LC2-13A and K-13 libraries were sequenced using an Ion 530 Chip with an Ion GeneStudio S5 System.

### 2.3 Genome assembly, annotation, and analysis

The sequence reads were analyzed and *de novo* assembly was performed using the CLC Genomic Workbench v.21.0.3. (Qiagen, Valencia, CA, USA). The genomes were annotated using the DDBJ Fast Annotation and Submission Tool (DFAST, https://dfast.nig.ac.jp/). Functional annotation was assigned using the dbCAN2 server (http://bcb.unl.edu/dbCAN2/index.php) [6]. Genome maps of *P. cisolokensis* LC2-13A and *X. composti* K-13 were obtained using CGView (http://cgview.ca/) [7].

## Ethics Statement

This research and analysis did not involve the use of human subjects or animal experiments.

## CRediT authorship contribution statement

**Ayaka Uke:** Conceptualization, Methodology, Data curation, Writing – original draft. **Chinda Chhe:** Conceptualization, Methodology. **Sirilak Baramee:** Methodology, Investigation, Data curation. **Chakrit Tachaapaikoon:** Methodology, Investigation, Writing – review & editing. **Patthra Pason:** Methodology, Investigation. **Rattiya Waeonukul:** Methodology, Investigation. **Khanok Ratanakhanokchai:** Methodology, Investigation. **Akihiko Kosugi:** Supervision, Writing – review & editing.

## Declaration of Competing Interest

The authors declare that they have no known competing financial interests or personal relationships that have or could be perceived to have influenced the work reported in this article.
